# Application of Multiple Unsupervised Models to Validate Clusters Robustness in Characterizing Smallholder Dairy Farmers

**DOI:** 10.1155/2019/1020521

**Published:** 2019-01-02

**Authors:** Devotha G. Nyambo, Edith T. Luhanga, Zaipuna O. Yonah, Fidalis D. N. Mujibi

**Affiliations:** ^1^Nelson Mandela African Institution of Science and Technology, P.O. Box 447, Arusha, Tanzania; ^2^USOMI Limited, P.O. Box 105086–00101, Nairobi, Kenya

## Abstract

The heterogeneity of smallholder dairy production systems complicates service provision, information sharing, and dissemination of new technologies, especially those needed to maximize productivity and profitability. In order to obtain homogenous groups within which interventions can be made, it is necessary to define clusters of farmers who undertake similar management activities. This paper explores robustness of production cluster definition using various unsupervised learning algorithms to assess the best approach to define clusters. Data were collected from 8179 smallholder dairy farms in Ethiopia and Tanzania. From a total of 500 variables, selection of the 35 variables used in defining production clusters and household membership to these clusters was determined by Principal Component Analysis and domain expert knowledge. Three clustering algorithms, K-means, fuzzy, and Self-Organizing Maps (SOM), were compared in terms of their grouping consistency and prediction accuracy. The model with the least household reallocation between clusters for training and testing data was deemed the most robust. Prediction accuracy was obtained by fitting a model with fixed effects model including production clusters on milk yield, sales, and choice of breeding method. Results indicated that, for the Ethiopian dataset, clusters derived from the fuzzy algorithm had the highest predictive power (77% for milk yield and 48% for milk sales), while for the Tanzania data, clusters derived from Self-Organizing Maps were the best performing. The average cluster membership reallocation was 15%, 12%, and 34% for K-means, SOM, and fuzzy, respectively, for households in Ethiopia. Based on the divergent performance of the various algorithms evaluated, it is evident that, despite similar information being available for the study populations, the uniqueness of the data from each country provided an over-riding influence on cluster robustness and prediction accuracy. The results obtained in this study demonstrate the difficulty of generalizing model application and use across countries and production systems, despite seemingly similar information being collected.

## 1. Introduction

Despite the high potential of livestock keeping, Ethiopia and Tanzania still suffer from low meat and milk production given that most livestock populations are dominated by low producing indigenous breeds [1, 2]. Smallholder farmers dominate the livestock keeping enterprise in Africa, accounting for about 50% of the total livestock production [3]. Dairy farming is an important source of income for smallholder farmers with high potentials for daily cash flow [4]. Majority of these smallholder producers have not reached their production potential in terms of yield and commercialization. However, data from a recent large-scale survey provides evidence that some farmers produce at a level well beyond the average production (PEARL data, 2016; unpublished). There are many constraints that contribute to the unreached potential, including lack of appropriate support in technologies and information dissemination.

Despite the constraints hindering smallholder dairy productivity, milk obtained from smallholder dairy farmers constitutes the bulk of supply available for sale in Eastern Africa [4]. Among the hindering factors in the provision of appropriate support to the dairy sector and evolvement of the dairy farmer beyond subsistence, is the lack of understanding of the production system these farmers are operating in. Characterization of farm typologies is a necessary first step in designing appropriate interventions that allow these farmers to improve farm output and performance. The characterization of production systems and identification of homogenous units that represent contemporary groups in management terms allow us to understand the specific attributes associated with drivers of productivity. This holds the key to unlocking the ingredients of household evolvement through proper planning, adoption, and utilization of appropriate improved technologies and critical policy support [5]. This study sought to provide a mechanism through which farmers that perform similar production activities or have similar production system attributes can be grouped together into production clusters that describe their organization, needs, and outputs.

Given the huge diversity of practices seen in smallholder farms, the need to form homogenous units that group farmers with near similar characteristics has been addressed in several studies. Primarily, this has been done by domain experts allocating farmers to various predetermined classes of farmers; defining their place in the production ecosystem, as well as statistical and machine learning approaches [6–10]. The latter approach involves use of various supervised and unsupervised algorithms to study, analyze, model, and predict trends in smallholder production systems. Recently, unsupervised learning algorithms have been applied in various studies to understand production systems [11, 12]. Some of the more popular unsupervised algorithms include hierarchical clustering, nonhierarchical clustering (K-means), unsupervised neural network algorithms (Self-Organizing Maps), Naïve Bayes and fuzzy clustering algorithms. However, despite their frequent use, unsupervised learning approaches suffer greatly from lack of consistency and predictability [13]. Various attempts have been made to overcome this weakness, including application of multiple algorithms to cluster farm data and select the one with highly homogeneous groups [14, 15].

In this study, three unsupervised machine learning (ML) models were applied to classify and study the characteristics of smallholder dairy production systems based on data obtained from baseline surveys in Ethiopia and Tanzania. The aim of the study was to identify the most robust approach to accurately assign diverse dairy farming households into homogenous production units that reflect the differences in production practice and performance.

## 2. Methodology

### 2.1. Dataset Preparation and Feature Selection

Data was collected under the PEARL (Program for Emerging Agricultural Research Leaders-Funded by the Bill and Melinda Gates Foundation through the Nelson Mandela African Institution of Science and Technology) project from June 2015 to June 2016 in Ethiopia and Tanzania. The total number of households surveyed was 3,500 for Tanzania and 4,679 for Ethiopia. Data collection was undertaken using questionnaires developed on the Open Data Kit (ODK) platform. Data quality checks included removal of erroneous data such as negative values, questionnaires whose total collection time was below a defined threshold (16 min), and data collected at night (survey start time beyond 7pm). The data cleaning process trimmed the datasets to 3317 and 4394 records for Tanzania and Ethiopia, respectively. From a total of 500 unique variables (features) available for analysis, a set of 46 variables were selected for inclusion in the cluster analysis based on their relevance to productivity and farmer evolvement.

*Feature Selection*. In order to identify the most unique features among the 46 variables, Principal Component Analysis (PCA) was undertaken to eliminate correlated variables. The top 21 features (based on the load score) with the lowest communality were then selected for further analysis. An additional 14 variables related to feeding systems and health management practices which are known to influence productivity in smallholder dairy farming were included based on expert domain knowledge, such that a total of 35 features were available for cluster analysis and farm type characterization (Table 1). As a prerequisite for clustering, missing values for continuous variables were identified and replaced with population means, while missing values for categorical values were replaced with mode value. The effect of location (study site) for each country was removed from the response variables by fitting a liner model (*y* = *μ* + *study*_*site*_ + *error*) and extracting adjusted values. Each quantitative variable was tested for normality and scaled to have a mean of zero and unit variance. Additionally, for each variable, outliers were identified as values above or below the bounds estimated using box plots. Outliers were removed to minimize bias and misclustering. Specifically, bias was minimized by applying the following filters.

The total number of cattle owned was restricted to a maximum of 50 per herd for Ethiopian farmers and a maximum of 30 per herd for Tanzanian farmers based on livestock densities [1, 2]. Some smallholder farmers held land holdings above 100 acres; all farmers with land holdings greater than 100 acres were removed. The maximum amount of milk sold by smallholder farmers was restricted to 100 liters per day, based on expert domain knowledge of the herd sizes and yield per cow. It was assumed that an extension officer could visit a farmer once each week. Any farmer who had more than 54 visits per year was considered an outlier.

### 2.2. Clustering Algorithms

Three unsupervised learning algorithms, fuzzy clustering, Self-Organizing Maps (SOM), and K-means, were used for cluster analysis. In the analysis, the number of groups (K) represented how many farm typologies (clusters) could be defined for each dataset. The number of clusters that best represented the data was determined using the Elbow method (where a bend or elbow in a graph showing decline of within cluster sum of squares differences as the number of clusters increases provides the best solution). Gap statistics and silhouette separation coefficients were used in preliminary analysis to validate the results from the Elbow method [16], while the Euclidean distance was used to assess cluster robustness. The Elbow method was found to be robust and subsequently used for the rest of the analysis. Given that the selected algorithms have various methods with different convergence rates, two methods for each algorithm were tested and those that minimized convergence time were selected. The final clustering methods used were (i) Fanny for fuzzy clustering [17], (ii) superSOM with batch mode [18], and (iii) Hartigan-Wong [19, 20] for K-means. Evaluation of the clustering algorithms was done by considering ranking consistency in the testing dataset, mean distance of observations from central nodes, and mean silhouette separation coefficients as well as accuracy of predicting observed values of select response variables using a model fitting the predicted clusters as fixed effects. Other evaluation criteria for the clustering algorithms were. Data analysis was done using both SAS version 9.2 (SAS Institute Inc., Cary, NC, USA) and R software (Kabacoff, 2011).

### 2.3. Clustering Models

**Self-Organizing Maps (SOM)** have been used to characterize smallholder farmers due to their ability to produce accurate typologies as explained by Nazari et al. [15] and Galluzzo [21]. The SOM algorithm calculates Euclidean distance by using (1) and the best matching unit (BMU) satisfying (2) [21, 22].

(1) $$\begin{array}{c} Distance=\sqrt{\overset{i=n}{\underset{i=0}{\sum }}{\left(vi-wi\right)}^{2}} \end{array}$$

where *v* and *w* are vectors in an n dimension Euclidean space relating to position of a member and neuron, respectively, and

(2) $$\begin{array}{c} \forall n_{i}\in S:diff\left(\;n_{winnerweight},v\;\right)\leq diff\left(\;n_{iweight},v\;\right) \end{array}$$

whereby* v* is any new weight vector, *n*_*winnerweight*_ is the current weight of the winning neuron, and *n*_*iweight*_ is a weight of any other *i*th neuron on the map.

**The K-means** algorithm has been widely used in nonhierarchical clustering and characterizing smallholder dairy farms [7, 8, 10]. Similar to SOMs, the algorithm uses Euclidean distance measures to estimate weights of data records. The algorithm is presented as. (3), with a segment of the Euclidean distance as in (1).

(3) $$\begin{array}{c} J=\overset{k}{\underset{j=1}{\sum }}\;\overset{n}{\underset{i=1}{\sum }}{\left|\;\left|\;x_{i}^{j}-c_{j}\;\right|\;\right|}^{2} \end{array}$$

where ||*x*_*i*_^*j*^ − *c*_*j*_||^2^ computes the Euclidean distance as in (1);* k* = number of clusters,* n*= number of observations, j = minimum number of clusters, i= minimum number of observations, *x*_*i*_ = Euclidean vector for any *i*th observation, and *c*_*j*_ = cluster center for any jth cluster.

**Fuzzy analysis (fanny method) **was selected based on its relatively short convergence time and good measures for clusters separation [17]. Various methods based on fuzzy models have been used for cluster analysis [23–26]. The fanny method adds a fuzzier and a membership value to the common K-means algorithm (see (3)). In addition, the model uses the Dunn coefficient and a silhouette separation coefficient for assessing the solution fuzziness and intercluster cohesion, respectively. The general equation for fuzzy clustering [27] is given in (4) and the Dunn definition of partitioning [28] is given in (5).

(4) $$\begin{array}{c} J=\overset{k}{\underset{i=1}{\sum }}\;\overset{n}{\underset{j=1}{\sum }}U_{ij}^{m}{\left|\;\left|\;x_{i}-c_{j}\;\right|\;\right|}^{2},\;1\leq m<\infty \end{array}$$

where k = number of clusters, n = number of observations,* i*= minimum number of clusters,* j*= minimum number of observations, *U*_*ij*_^*m*^ =membership coefficient, *x*_*i*_ = Euclidean vector for any *i*th observation, and *c*_*j*_ = cluster center for any jth cluster. Given (4), the Dunn definition of partitioning is given by

(5) $$\begin{array}{c} F_{k}\left(\;U\;\right)=\left(\;\frac{1}{n}\;\right)\overset{k}{\underset{i=1}{\sum }}\;\overset{n}{\underset{j=1}{\sum }}U_{ij}^{m} \end{array}$$

### 2.4. Cluster Validation and Prediction Accuracy

Production clusters outputted from the clustering algorithms were validated in three ways: (1) assessment of cluster robustness, (2) comparison of the cluster membership reallocation (differential allocation of households to clusters for training and testing datasets), and (3) evaluation of the proportion of variation explained by the clusters.

Validation of cluster robustness was first undertaken by comparing three metrics: total within sum of square differences, mean Euclidean distance of observations from the cluster nodes, and the silhouette separation coefficients. Based on these parameters, the most suitable clustering model was identified. In the second stage of validation, the ability of the clustering models to allocate the same group of households into clusters in both training and testing datasets was tested. If all cluster members are colocated in one cluster in training and testing datasets, the reranking is 0 (the rank correlation between the two clusters is 1), and the model would be deemed the most accurate and robust. Parameters considered for evaluation were correlation coefficient, AIC, and residual deviance. The third stage of validation involved fitting linear (or logistic as appropriate) regression models with a set of fixed effects on milk yield, sales, and choice of breeding method. The first model (see (6) and (9)) included the clusters as one of the fixed effects while a second model did not include the clusters (see (7) and (10)). The difference in variance between the two models represented the proportion of total variance in the response variable accounted for by the clusters. The logistic model for choice of breeding method was fitted with only the cluster of production (see (8)) for Ethiopian data while two models were fitted for Tanzania (see (11) and (12)). In preliminary analysis, a model fitted with cluster of production yielded best fit results in the Ethiopia dataset and very low variances as a result of under fitting for the Tanzania dataset. For that reason, two models were fitted for Tanzania and one for Ethiopia to predict the binary variable. Class labels for the logistic regression were 0 and 1 for choice of bull method and Artificial Insemination, respectively. For assessing prediction accuracy, one-third of the records for the response variables were removed so that they could be predicted. The predicted values were correlated with the actual values to obtain an estimate of the prediction accuracy. These latter prediction accuracies were compared with those obtained in the previous validation step to help evaluate the algorithms' consistency and clusters' robustness.

(6) yi=xe∗γe+ce+ee

(7) yi=xe∗γe+ee

The logistic model used to predict choice of breeding method is shown in

(8) $$\begin{array}{c} y_{j}=c_{e}+e_{e} \end{array}$$

For Tanzania, predictive models were given by

(9) yi=xt∗γt+lt+σt+ct+et

(10) yi=xt∗γt+lt+σt+et

And choice of breeding method was given by (see (11) and (12))

(11) yj=xt+γt+ct+et

(12) yj=xt+γt+et

where y_i_ is milk yield or milk quantity sold and y_j_ is choice of breeding method. For the Ethiopia models, c_e_ is cluster of production, e_e_ is the error term, x_e_ is experience in dairy farming, and *γ*_*e*_ is years of schooling. For the Tanzania models, c_t_ is cluster of production, e_t_ is the error term, x_t_ is experience in dairy farming, *γ*_t_ is years of schooling, l_t_ is total land size, and *σ*_t_ is area under fodder production.

For all model validation steps, prediction accuracies were obtained by developing the clustering model in a training dataset (70% of all records) and the resulting model reapplied to a testing dataset (remaining 30%). The model with the least reallocation of households between clusters for the training and testing datasets was considered the most robust. Rank analysis using the spearman correlation coefficient was used to evaluate the level of household reallocation between clusters.

## 3. Results 

### 3.1. Clustering

**Figure 1:**
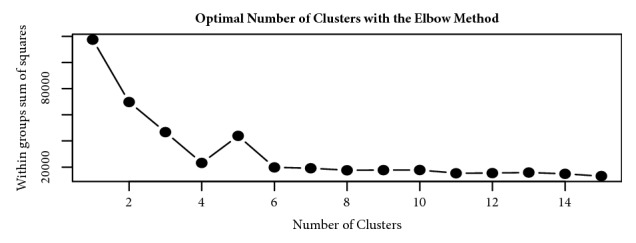
Graph showing four optimal clusters for the Ethiopia dataset.

**Figure 2:**
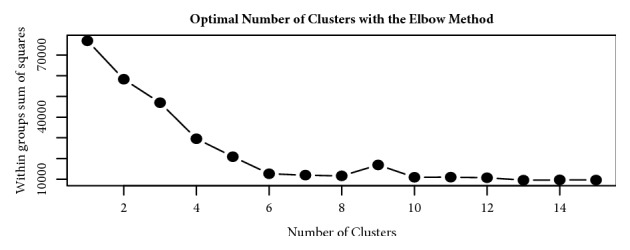
Graph showing six optimal clusters for the Tanzania dataset.

Based on the Elbow method, a four cluster solution was found to be optimal for the Ethiopia dataset and was fitted in the clustering models (Figure 1). The SOM and K-means algorithms clustered the households in the Ethiopia dataset into four groups, while the fuzzy model assigned all households into three clusters, with no members in the fourth cluster.  Table 2 shows the cluster densities for each algorithm. For Tanzania, six clusters were defined based on the Elbow method (Figure 2). However, at K=6, the fuzzy model had highly fuzzy cluster memberships of 0.09 and 0.18 for each member. Such low membership values imply an unstable cluster solution. The fuzzy model was therefore discarded for the Tanzania dataset and analysis proceeded with the K-means and Self-Organizing Maps (SOM) algorithms. Cluster densities associated with the six clusters are provided in Table 3.

**Figure 3:**
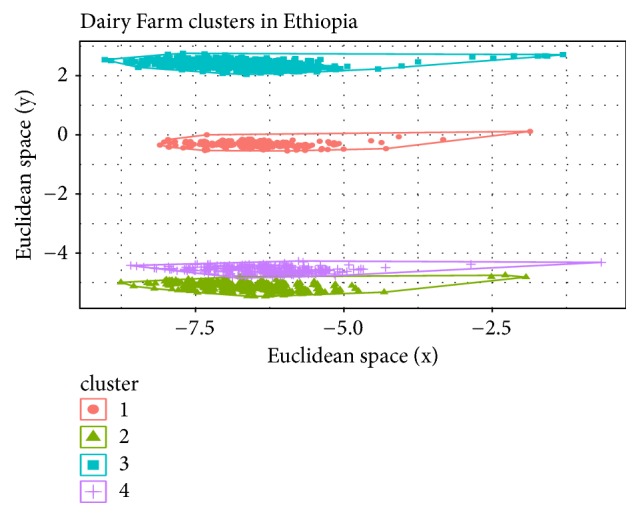
Household allocation to four clusters using the K-means model for Ethiopia dairy farmers.

**Figure 4:**
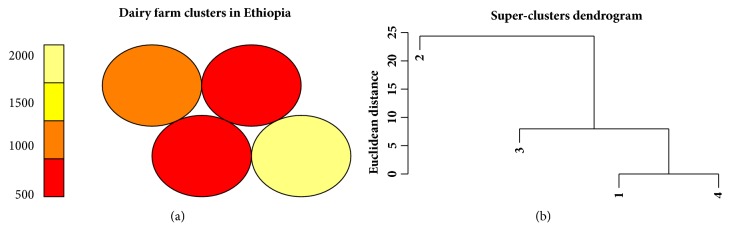
Node counts for household clusters derived using the SOM model for Ethiopia (a) and dendrogram for super clusters (b).

**Figure 5:**
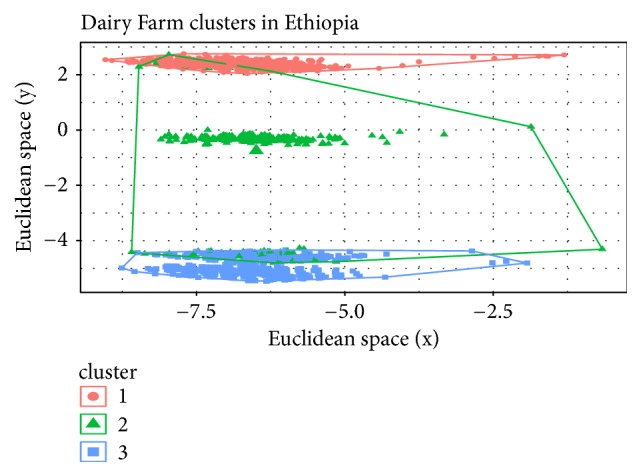
Household allocation into three clusters using the fuzzy model for Ethiopia dairy farmers.

For the Ethiopian data, cluster densities given in Table 2 indicate the presence of one unchanging cluster for both K-means and SOM models (with the exact same list of 487 members). The number of members in the other clusters varied, indicating households being reassigned to different clusters. Figures 3, 4, and 5 represent the cluster visualization for each algorithm in the Ethiopia dataset. Clusters obtained using K-means were well separated and showed significant intracluster adhesion (Figure 3), while spatial distribution of SOM clusters (Figure 4) indicated significant overlap between two of the 4 clusters (clusters in red). Cluster densities for Tanzania are displayed in Table 3.

Figures 4(a) and 4(b) are a heatmap representation of cluster densities and dendrogram from the SOM model, respectively.  Figure 4(a) shows counts of households within clusters while Figure 4(b) indicates cluster relationship and separation. The numbers on the colored plane indicate number of members in each cluster. Two clusters had equal number of farmers (shown in red color) and on the dendrogram these are categorized as clusters 1 and 4. These two clusters seemingly had few differentiating features since they originate from the same parent node. This phenomenon can also be observed in Figure 3 for the K-means model (clusters 2 and 4). These clusters appear to be joined into one cluster in the fuzzy model (cluster 3 in Figure 5). The fuzzy model resulted in 3 clusters, each with a significant number of outliers (Figure 5). The outliers were however more pronounced for cluster 2 than clusters 1 and 3.

Presence of the outliers and cluster overlap in the fuzzy model was supported by a low value of the Dunn coefficient (0.3014) which corresponds to a high level of fuzziness.

Based on the results obtained, the cluster composition parameters related to intercluster adhesion and intracluster cohesion indicated that clusters from the K-means model were better separated (higher mean silhouette value) and more compact (lower mean distance from central node) than in the other models for Ethiopia (Table 4).

**Figure 6:**
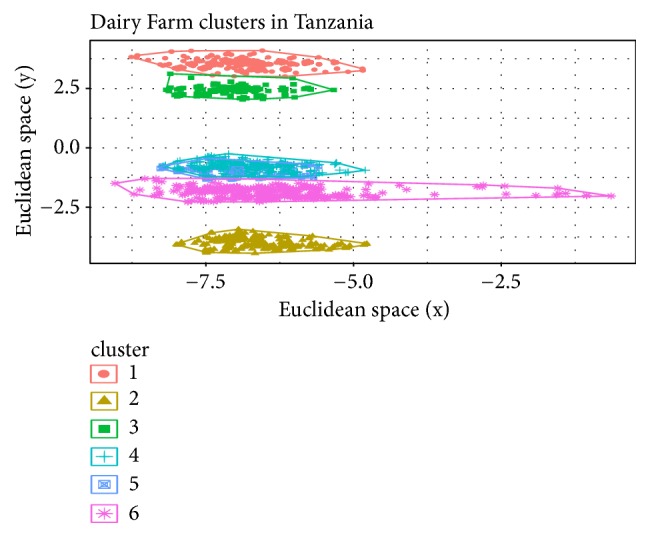
Household allocation into six clusters using the K-means model for Tanzania dairy farmers.

**Figure 7:**
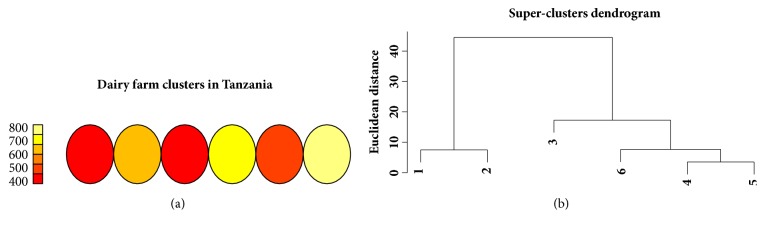
Node counts for household clusters derived using the SOM model for Tanzania (a) and dendrogram for super clusters (b).

For Tanzania, the mean silhouette separation coefficients were not significantly different (0.66 and 0.64 for K-means and SOM, respectively) as shown in Table 5. However, there was a tendency for the SOM to have better defined clusters given its lower within cluster sum of squares as well as lower mean distance from central node. The spatial distribution is illustrated in Figures 6 and 7.

For Tanzania clusters' separation and intactness can be observed through Figures 6 and 7. No significant difference can be observed with regard to the intercluster adhesion between K-means and SOM (Table 5).

Figure 6 shows clusters visualization from the K-means model for Tanzania dataset. Cluster 4 and 5 overlap and are in close proximity to cluster 6, indicating that they have few differentiating characteristics. This overlapping is equally observed in the SOM model (Figure 7).

The numbers on the colored bar in Figure 7(a) indicate densities of members in each cluster. There are only four well separated clusters based on density (from left: red, orange, yellow, and light gold). However, the dendrogram (Figure 7(b)) shows that three clusters, branching from the same node, which also are also seen as the overlapping clusters (clusters 4, 5, and 6) in the K-means plot (Figure 6)

### 3.2. Cluster Validation

#### 3.2.1. Cluster Membership Reranking

Ranking correlation was used to study the levels of household relocation for the training and testing datasets. Generally, the clustering models applied to the Ethiopia dataset indicated low membership relocation.  Table 6 summarizes the results for Ethiopia where, despite a lower Akaike Information Criteria (AIC) estimate, the fuzzy model had the highest number of members reallocated to other clusters (32%) compared to the K-means and SOM. The high correlation coefficients for SOM and K-means indicate lower reallocation of cluster members. In contrast, results from Tanzania indicated very high reranking of cluster membership between training and testing datasets (Table 7).

#### 3.2.2. Prediction Accuracy

Tables 8 and 9 summarize the results for predicting missing values for milk yield, sales, and breeding choice. Results for Ethiopia dataset indicate that model fitting fixed effects of clusters derived from the fuzzy model had higher accuracies for peak milk yield (0.77), milk sales (0.48), and probability of choosing AI (0.55) as shown in Table 8, while for Tanzania, higher accuracies were obtained for milk production and sales (0.46 and 0.41) while fitting clusters were obtained from the K-means model (Table 9).

For the Tanzania dataset, clusters from the K-means model achieved high prediction accuracies for both milk yield and sales (at 46% and 41%, respectively). However, the K-means clusters had lower prediction accuracy for choice of breeding method (29%). Clusters from the SOM model performed poorly on the quantitative traits but had higher probability (46%) for correctly assigning the choice of breeding method.

#### 3.2.3. Cluster Variances

In order to assess whether the clusters defined by the various algorithms reflect differences in production characteristics between households, we evaluated the variance accounted for by these cluster on select performance measures. For Ethiopia, total variance was 1.015 and 0.988 for milk yield and sales, respectively, while in Tanzania, the total variance was 1.076 and 1.09 for milk yield and sales, respectively. The differences between residual variances for two linear models (see (6) versus (7) for Ethiopia and (9) versus (10) for Tanzania) were significant (p < 0.00001). Results show that, for Ethiopia data, the fuzzy model clusters accounted for 89% and 70% of the total variance in milk yield and milk sales, respectively. On the other hand, the K-means clusters accounted for 71% and 65% of the total variation in milk yield and milk sales, respectively. Tables 10 and 11 summarize the proportion of variances accounted for by the clusters for each clustering model.

## 4. Discussion

### 4.1. Characterization of Smallholder Farmers

Unsupervised learning models have been used to characterize smallholder farmers despite the fact that these models lack consistency and are highly unpredictable [13]. In this study, the performance of three commonly used algorithms for clustering farming households; namely, K-means, fuzzy, and SOM were compared. A set of validation criteria to assess the robustness of the defined clusters is proposed. This approach is seldom used for similar studies.

In Africa, smallholder farming systems have been characterized using common hierarchical and nonhierarchical clustering algorithms. Work done by Mburu et al. [29], Bidogeza* et al*. [30], Dossa* et al*. [10], and Kuivanen* et al*. [7, 8] utilized the ward and K-means methods to define clusters for smallholder households. In addition to the machine learning approaches, use of expert knowledge to validate cluster based characterization is highly recommended [7, 8]. In some studies, the local knowledge has been used in a participatory approach to accurately estimate farm types. Furthermore, complex clustering approaches have also been explored in studying smallholder farm types as done by Salasya & Stoorvogel [23], Pelcat* et al*. [31], Galluzzo [21], and Paas & Groot [12]. These studies present use of fuzzy clustering, Neural Networks, and Naïve Bayes algorithms, respectively. Although all clustering assigns farmers into some types, the fuzzy clustering presents a soft clustering approach where a farm can belong to more than one farm type or none [31]. However, from the analyzed previous researches clustering models' robustness and their ability to predict farm types remains uncharted. Following up on Goswami* et al*. [5] study of smallholder farmers needs to be subjected into formulation of predictive farm types. As such, evolvement of farmers in the homogeneous groups can be predicted because the clusters' stabilities are known.

### 4.2. Clustering Algorithms Evaluated

The determination of putative number of clusters that best define the data (K) presents the foremost need in cluster analysis. Bad estimates of K may result into unstable clusters and presence of many members appearing as outliers. Since the goal is to obtain highly homogeneous groups, the within group sum of square difference is commonly used to evaluate how compact the clusters are. We adopted recommendations given by Kassambara [16] and employed the Elbow, Gap statistics, and average silhouette methods to assess the best K for the datasets. The Elbow and Gap statistics estimate a value of K that minimizes the within groups sums of square (WSS) differences such that any additions to the estimated value of K will not significantly change the WSS. Since the study goal was to arrive at highly homogeneous groups, the measure of within sum of square differences seemed most important. However, a common method to estimate optimal number of clusters from other studies is to try out different values of K while observing the silhouette separation or manual inspection of dendrogram produced in hierarchical clustering [15, 16]. While the Elbow method and Gap statistics use within groups sum of square differences, the silhouette method compares the average clusters separation.

The application of the three separate algorithms revealed differences in their performance based on data type and structure. Where observations were highly identical, soft clustering (fuzzy model) failed to categorize the records into appropriate number of clusters. The fuzzy model allocated households into only 3 clusters despite four clusters being determined as appropriate for the Ethiopia dataset (Figure 5). The other models converged at 4 clusters (Figures 3 and 4). Similarly, for the Tanzanian dataset, the fuzzy model could not converge even after many iterations. It would appear that the fuzzy model is best suited to situations where data is highly heterogeneous. Otherwise it does not lend itself well to cluster identification.

Balakrishnan (1994) compared K-means and SOM algorithms in cluster identification within specific criterion of intracluster similarity and intercluster differences. In addition, the dataset had known cluster solutions; so, the only target was to find out performance differences between the two algorithms. Results indicated that the K-means algorithm had good performance over the SOM algorithm. Mingoti & Lima [32] compared K-means and SOM models' performance by using smallholders' farm data. Results indicated that K-means were more robust. In this study, the SOM performed poorly compared to the fuzzy and K-means for the Ethiopia dataset having higher within cluster dispersion, as well as lower separation between clusters. For the Tanzania dataset, the SOM performed similarly as the K-means algorithm. Results from our study show that the performance of SOM is concordant with that of Nazari* et al*. [15] who characterized dryland farming systems. In contrast to observations by Mingoti & Lima [32], the fuzzy model used in their study failed spectacularly for both datasets. This reinforces observations by Xu [33] who concluded that the performance of clustering algorithms is subject to the nature of data and area of application. More studies need to be undertaken to see how the fuzzy algorithm can be best adapted to farming datasets.

### 4.3. Cluster Membership Reallocation and Prediction Accuracy

A good clustering model should be able to repeatedly allocate a majority of households into the same clusters, even when the volume of data changes. In order to be sure that our model definitions represented a collection of the most important features that describe each cluster, we tested the ability of the models to redefine the same clusters between training and testing datasets. This strategy aligns well with Xu [33], who recommends that a good clustering model should have the ability to deal with new data cases without the need to relearn. The spearman rank correlation was used to measure the degree of reranking. For the Tanzania data, the SOM model provided the best cluster allocation that minimizes reranking. The rank correlations seen in Tanzania were very low for both the K-means and SOM models. Given the above premise and the spectacular failure of the fuzzy model in Tanzania, a pattern emerges to suggest a fundamental problem with the Tanzanian dataset rather than issues to do with model suitability. It is possible that there is no significant differentiation between households in Tanzania and the extreme homogeneity proves a challenge because each household can be allocated to any cluster. Such a scenario could occur due to flawed data collection strategies. We suspect that, due to requirements to finalize data collection within set timelines, groups of farmers were interviewed collectively while data was entered as if it were for an individual farmer.

The fuzzy model in Ethiopia had the best fit, indicated by the lowest AIC value despite higher membership reallocation. Given a standard prediction problem, this would be the best model for the data. This is also corroborated by the fact that the variance accounted for by the clusters was also highest for the fuzzy model. However, given that our intention is to maximize correct reassignment of individuals into clusters, the K-means and SOM models would be preferred for household membership allocation.

Three response variables (milk yield, sales, and choice of breeding method) were selected for the prediction exercise because of their vital role in smallholder dairy farm evolvement. They generally represent the commercial orientation of a smallholder farm. Evaluation of prediction accuracies for selected response variable indicated a very different scenario from the clustering problem. When the clusters were included in the models to predict milk yield, sales, or breeding method, the fuzzy model-derived clusters had the highest prediction accuracies compared to K-means and SOM clusters for Ethiopia data. For Tanzania data, the SOM model clusters yielded the best prediction accuracies for the binary trait, choice of breeding method, while K-means model performed the best for the quantitative traits. However, the prediction accuracies for the Tanzania data were low, underscoring the earlier assertions about data structure and integrity. Given the predictive power of the clusters on select response variables, the fuzzy clustering model performed the best, with defined clusters accounting for significantly higher variations in the response variable than other clustering models.

Based on the results from Ethiopia, where all the models could be evaluated, it would seem that model choice depends on the problem that needs to be solved. For a clustering problem, where the intention is to obtain robust membership allocation, then the K-means algorithm would be the most appropriate, to ensure maximal homogeneity within clusters. The use of this model would minimize reranking when applying the model to new datasets without need for new learning. However, in the event that clusters are to be used in prediction models, the fuzzy algorithm would be the best for clusters definition.

## 5. Conclusion

The goal of the reported study was to identify the most robust approach to correctly classify diverse households into homogenous groups of farmers with similar production systems and management activities. The reason for the characterization was to use the defined groups in order to design interventions and strategies that facilitate the evolvement of smallholder dairy farmers beyond subsistence in Ethiopia and Tanzania. Results from this study demonstrate the use of unsupervised learning models in cluster definition for smallholder dairy farmers as well as strategies to assess the models' suitability and cluster robustness. Performance varied across the tested models, underscoring the need to find an appropriate method depending on data structure and questions being answered. The results obtained from this study are a necessary first step in understanding smallholder farmer production systems and the study of household evolvement from subsistence to full commercial orientation.

## Figures and Tables

**Table 1:** Features used in cluster analysis.

**S/No**	**Feature Name**	**Type**	**Range**
**1**	Exclusive grazing in dry season	Boolean	0(no) or 1(yes)
**2**	Exclusive grazing in rainy season	Boolean	0(no) or 1(yes)
**3**	Mainly grazing in dry season	Boolean	0(no) or 1(yes)
**4**	Mainly grazing in rainy season	Boolean	0(no) or 1(yes)
**5**	Mainly stall feed in dry season	Boolean	0(no) or 1(yes)
**6**	Mainly stall feed in rainy season	Boolean	0(no) or 1(yes)
**7**	Use of concentrates	Discrete	1 – 12 (months)
**8**	Watering frequency	Discrete	0 – 4
**9**	Distance to water source	Continuous	0 – 15
**10**	Total land holding	Continuous	0 – 100
**11**	Area under cash cropping	Continuous	0 – 10
**12**	Area under food cropping	Continuous	0 – 83.25
**13**	Area under fodder production	Continuous	0 - 80
**14**	Area under grazing	Continuous	0 - 13
**15**	Number of employees	Discrete	1 - 10
**16**	Number of casual labors	Discrete	1 – 10
**17**	Vaccination frequency	Discrete	0 – 6
**18**	Deworming frequency	Discrete	0 – 5
**19**	Self-deworming service	Boolean	0(no) or 1(yes)
**20**	Membership in farmer groups	Discrete	0 – 5
**21**	Experience in dairy farming	Discrete	1 - 50
**22**	Years of schooling	Discrete	0 – 21
**23**	Preferred breeding method	Boolean	0 (bull) or 1(artificial insemination)
**24**	Distance to breeding service provider	Continuous	0 - 100
**25**	Frequency of visit by extension officer	Discrete	1 – 54
**26**	Herd size	Discrete	1 – 50
**27**	Number of milking cows	Discrete	1 – 20
**28**	Number of exotic cattle	Discrete	1 - 48
**29**	Number of sheep	Discrete	1 - 80
**30**	Peak milk production for the best cow	Continuous	1 – 40
**31**	Amount of milk sold in bulk	Continuous	1 – 100
**32**	Liters of milk sold	Continuous	1 – 100
**33**	Distance to milk buyers	Continuous	1 – 37
**34**	Total crop sale	Continuous	0 – 21000 (Birr), 0 – 950000 (Tsh)
**35**	Distance to market	Continuous	1 – 8

**Table 2:** Cluster densities (number of households allocated to the cluster) for the Ethiopia dataset.

**Cluster**	**K-means model**	**SOM model**	**Fuzzy model**
**1**	342	487	2673
**2**	875	2084	411
**3**	2689	1217	1309
**4**	487	605	

**Table 3:** Cluster densities (number of households allocated to the cluster) for the Tanzania dataset.

**Cluster**	**K-means model**	**SOM model**	**Fuzzy model**
**1**	811	1180	2506
**2**	452	952	811
**3**	374	203	
**4**	616	295	
**5**	372	516	
**6**	692	171	

**Table 4:** Cluster composition parameters (intercluster adhesion and intracluster cohesion) for Ethiopian households.

Model	No. Clusters	Within sum of square	Mean distance from central nodes	Mean silhouette separation
**K-means model**	4	20758	0.74	0.66
**SOM model**	4	23178	0.92	0.51
**Fuzzy model**	3	21655	0.89	0.56

**Table 5:** Cluster composition parameters (intercluster adhesion and intracluster cohesion) for Tanzania households.

Model	No. Clusters	Within sum of square	Mean distance from central nodes	Mean silhouette separation
**K-means model**	6	12628	2.1	0.66
**SOM model**	6	11772	1.7	0.64

**Table 6:** Cluster model parameters and ranking accuracy (membership reallocation) based on spearman rank correlation for the Ethiopia dataset.

Model	AIC	Residual deviance	Ranking accuracy (r)
**K-means model**	102	2.7e^∧^-2	0.85
**SOM model**	102	2.8e^∧^-2	-0.88
**Fuzzy model**	68.09	9.35e^∧^-2	0.68

**Table 7:** Cluster model parameters and ranking accuracy (membership reallocation) based on spearman rank correlation for the Tanzania dataset.

Model	AIC	Residual deviance	Ranking accuracy (r)
**K-means model**	200	0.001	-0.21
**SOM model**	200	0.006	0.39

**Table 8:** Estimates of prediction accuracy for models fitting cluster of production for milk yield, milk sales, and choice of breeding method in Ethiopia.

	Accuracy of prediction (r)		0 ≤ p ≤ 1
Algorithm/Response Variable	Milk yield	Milk sold	Preferred breeding method
**K-means**	0.68	0.40	0.54
**SOM **	0.66	0.38	0.54
**Fuzzy**	0.77	0.48	0.55

**Table 9:** Estimates of prediction accuracy for models fitting cluster of production for milk yield, milk sales, and choice of breeding method in Tanzania.

	Accuracy of prediction (r)		0 ≤ p ≤ 1
Algorithm/ Response Variable	**Milk yield**	**Milk sold**	**Preferred breeding method**
K-means	**0.46**	**0.41**	**0.29**
SOM	**0.32**	**0.31**	**0.46**

**Table 10:** Proportion of variance accounted for by cluster of production in Ethiopia.

	Fitted model	Total Variance*∗*	Residual variance	-2log likelihood	P value	Variance accounted for by cluster
**K-means**		**Milk yield**				
	Model with cluster	1.015	0.239	1867.4	<0.00001	73%
	Model without cluster		0.977	3718.4		
		**Milk sales**				
	Model with cluster	0.988	0.222	1770.1	<0.00001	54%
	Model without cluster		0.76	3388.6		

**SOM**		**Milk yield**				
	Model with cluster	1.015	0.283	2091.8	<0.00001	68%
	Model without cluster		0.977	3718.4		
		**Milk sales**				
	Model with cluster	0.988	0.258	1969.8	<0.00001	51%
	Model without cluster		0.76	3388.6		

**Fuzzy**		**Milk yield**				
	Model with cluster	1.015	0.074	337	<0.00001	89%
	Model without cluster		0.977	3718.4		
		**Milk sales**				
	Model with cluster	0.988	0.073	319.4	<0.00001	70%
	Model without cluster		0.76	3388.6		

*∗*Data scaled to have unit variance and mean of zero.

**Table 11:** Proportion of variances accounted for by cluster of production in Tanzania.

	Fitted model	Total variance*∗*	Residual Variance	-2log likelihood	P value	Variance accounted for by cluster
**K-means**		**Milk yield**				
	Model with cluster	1.076	0.0027	-2981	<0.00001	71%
	Model without cluster		0.771	2584.2		
		**Milk sales**				
	Model with cluster	1.09	0.018	-1084.3	<0.00001	65%
	Model without cluster		0.723	2520		

**SOM**		**Milk yield**				
	Model with cluster	1.076	0.294	1633	<0.00001	44%
	Model without cluster		0.771	2584.2		
		**Milk sales**				
	Model with cluster	1.09	0.228	1381.6	<0.00001	45%
	Model without cluster		0.723	2520.2		

*∗* indicates data scaled to have unit variance and mean of zero.

## Data Availability

The data used to support the findings of this study are available from the corresponding author upon request.
